# Total Knee Replacement with an Uncemented Porous Tantalum Tibia Component: A Failure Analysis

**DOI:** 10.3390/ma15072575

**Published:** 2022-03-31

**Authors:** Samo K. Fokter, Nenad Gubeljak, Esther Punzón-Quijorna, Primož Pelicon, Mitja Kelemen, Primož Vavpetič, Jožef Predan, Luka Ferlič, Igor Novak

**Affiliations:** 1 Department of Orthopaedics, University Medical Centre Maribor, Ljubljanska 5, 2000 Maribor, Slovenia; igornovak@yahoo.com; 2 Faculty of Mechanical Engineering, University of Maribor, Smetanova 17, 2000 Maribor, Slovenia; nenad.gubeljak@um.si (N.G.); jozef.predan@um.si (J.P.); luka.ferlic@um.si (L.F.); 3 Department of Low and Medium Energy Physics F2, Jožef Stefan Institute, Jamova 39, 1000 Ljubljana, Slovenia; esther.punzon@gmail.com (E.P.-Q.); primoz.pelicon@ijs.si (P.P.); mitja.kelemen@ijs.si (M.K.); primoz.vavpetic@ijs.si (P.V.)

**Keywords:** total knee arthroplasty, uncemented, cementless, trabecular metal, porous tantalum, tibial component, cementless baseplate fracture

## Abstract

Porous tantalum has been extensively used in orthopaedic surgery, including uncemented total knee arthroplasty (TKA). Favourable results were reported with earlier monobloc tibial components and the design evolved to modular implants. We aimed to analyse possible causes for extensive medial tibia bone loss, resulting in modular porous tantalum tibia baseplate fracture after primary TKA. Retrieved tissue samples were scanned with 3 MeV focused proton beam for Proton-Induced X-ray Emission (micro-PIXE) elemental analysis. Fractographic and microstructural analysis were performed by stereomicroscopy. A full 3D finite-element model was made for numerical analysis of stress–strain conditions of the tibial baseplate. Histological examination of tissue underneath the broken part of the tibial baseplate revealed dark-stained metal debris, which was confirmed by micro-PIXE to consist of tantalum and titanium. Fractographic analysis and tensile testing showed that the failure of the tibial baseplate fulfilled the criteria of a typical fatigue fracture. Microstructural analysis of the contact surface revealed signs of bone ingrowth in 22.5% of the surface only and was even less pronounced in the medial half of the tibial baseplate. Further studies are needed to confirm the responsibility of metal debris for an increased bone absorption leading to catastrophic tibial tray failure.

## 1. Introduction

Arthroplasty is the method of choice for the treatment of end-stage knee osteoarthritis (OA). In the UK, 90% of the procedures are performed as total knee arthroplasties (TKAs), and 86% of all primary knee arthroplasties are fixed with polymethylmethacrylate (PMMA) or bone cement [1]. Cemented TKAs with implant survivorship greater than 95% are regarded as the gold standard for patients undergoing knee replacement [2]. However, uncemented TKAs have been introduced in order to overcome higher rates of revision of cemented TKAs in younger and more active patients due to aseptic loosening, which is the known reactive process to wear debris from the polyethylene (PE) insert. In theory, uncemented implant fixation would enable direct osteointegration, reduce debris wear, and eliminate systemic complications associated with PMMA impaction [3]. Unfortunately, many of the earlier designs have failed because of uncemented metal-back patellar resurfacing and an increased risk of aseptic loosening of the tibia component [4].

Uncemented TKA gained renewed popularity after 1999 with the introduction of trabecular metal, which is a 75–80% porous tantalum (NexGen, Zimmer, Inc., Warsaw, IN, USA) with a high coefficient of friction [5]. Porous tantalum acetabular components and intervertebral cages reportedly achieved good short-term results [6,7]. The first commercially available porous tantalum design for use in knee arthroplasty was the monoblock tibia tray with two hexagonal pegs and a fixed articular ultra-high molecular weight polyethylene (UHMWPE) surface. Due to this, it eliminated backside wear and potentially reduced polyethylene debris. In combination with the cementless Co-Cr femoral component with fibre metal mesh ingrowth surface, the system has shown excellent mid-term clinical and registry-based outcomes [2,8]. Radiostereophotogrammetric analysis has shown porous tantalum components subsidence during the initial 3 months that stabilised afterward. This was regarded as beneficial for uncemented fixation [9]. In 2007, the manufacturer changed monoblock tibia component design to porous tantalum-coated titanium-alloy tray, allowing the attachment of separate modular UHMWPE inserts of different thicknesses. Additionally, a small circular peg (central boss) was added to the central anterior portion of the baseplate to cover the bore for a lock-down screw, which is used to additionally stabilise thicker UHMWPE inserts. Contrary to the fair amount of literature dealing with the monoblock design, only a very limited number of short to mid-term studies are available regarding the modular version of the porous tantalum tibia baseplate [10,11]. Hanzlik et al. reported a higher bone ingrowth of modular compared to monoblock trays [12]. However, Scully et al. recently reported a catastrophic modular tibia baseplate failure 4 years after implantation and found incomplete biologic ingrowth, suboptimal component sizing, and elevated patient body weight as possible reasons for revision [13].

The aim of the present study was to analyse other possible reasons for a tilting migration comprising subsidence necessitating an early revision of uncemented porous tantalum posterior-stabilised (PS) modular tibia component.

## 2. Materials and Methods

Institutional review board approval was obtained for the study. The patient in whom revision TKA was performed gave informed consent, and tissue samples taken during revision surgery were available for in-depth analysis. With primary surgical treatment, the patient received cementless posterior-stabilised TKA with a tibial baseplate made of Ti6Al4V alloy covered with porous tantalum for primary stability and secondary bone ingrowth (NexGen© LPS-Flex Trabecular Metal™ tibial tray, Zimmer, Warsaw, IN, USA). The patient was revised 3.6 years after primary TKA because of knee pain, aseptic loosening of the tibial component, recurrent varus deformity, and eventually fracture of the tibial baseplate.

Tissue samples were obtained during the revision surgery and sent for cultures and histological examination. The biopsy tissue was stained to delimit the borders with the Davidson Marking System^®®^ (DMS) in blue colour (#3408-5, Bradley Products, Inc.Bloomington, MN, USA). After that, samples were fixed in 10% neutral-buffered formalin and placed in paraffin blocks with the standard process using a Excelsior^TM^ AS Tissue Processor (Thermo Fisher Scientific, Waltham, MA, USA) and Tissue Embedding System TES99 (MEDITE Cancer Diagnostics, Inc. Chicago, IL, USA). In order to compare the results, consecutive slices were cut from the paraffin blocks. Then, 2 μm thick tissue slices were layered on the object glass and stained with standard haematoxylin and eosin staining technique. Finally, coverslips were mounted over the tissue specimens on the slides, and the samples were analysed by an experienced pathologist. Slices of 20 µm thickness were cut, sandwiched between two aluminium frames with 1 µm thickness mylar windows, and scanned with 3 MeV focused proton beam for Proton-Induced X-ray Emission (micro-PIXE) elemental analysis. The analysis was performed at the high-energy focused ion beam facility at Jožef Stefan Institute (JSI) with a proton beam of approximately 1 × 1 µm^2^ size and up to 300 pA analytical current. The induced X-rays were collected with an SDD detector (RaySpec Limited, High Wycombe, UK) placed 135 degrees scattered angle from the incoming beam. The resulting quantitative elemental maps of selected tissue areas reveal the eventual presence of Ta containing debris and its precise elemental composition [14,15].

Fractographic analysis was performed on a Keyence VHX 7000 (Keyence Corporation, Osaka, Japan) stereomicroscope with up to 6000 times magnification. Macroscopic examination and microstructural analysis of the metallographic samples from tibial baseplate were also performed by light microscopy (Keyence VHX-7000 digital microscope with the image analysis system, Keyence Corp., Osaka, Japan).

Removed implants were measured, and a finite element (FE) model was made for mechanical analysis. The model was made as a 3D solid body with deformable finite elements and consisted of 1.25 million 4-node tetrahedron FEs. The finite element analysis (FEA) was done on the 3D FE model by Abaqus Simulia Explicit 6.13 software (Dessault Systems, Vélizy-Villacoublay, France). The model has been used for numerical analysis of stress–strain conditions and simulations of the origin of the fatigue crack initiation and propagation. A full 3D FE model was used to get displacement and stress fields of the tibial baseplate with the patient standing on one foot, and the central part of each half of the tibial tray was loaded with the patient’s half body weight (440 N). The medial tibial condyle bone loss was simulated with the medial part of the tibial tray left unsupported.

The process for sample preparation for experimental determination of tensile mechanical properties of Ti-alloy has been previously described [16]. Briefly, mini tensile specimens were cut from the retrieved component by the spark wire erosion technique. Tensile testing was performed on an Instron Control panel 800 and Instron 1255 servo hydraulic testing machine certified by the United Kingdom Accreditation Service (UKAS) Cal. No. 42072-1999 and National Building and Civil Engineering Institute No. 15-0001-A. During the test, the resulting load vs. displacement was recorded. Displacement was measured stereo-optically by the Aramis system (GOM GmbH, Braunschweig, Germany). The engineering tensile stress–strain curve has been determined according to the Standard European Norm (SIST EN 1002-1:2002).

## 3. Results

**Figure 1:**
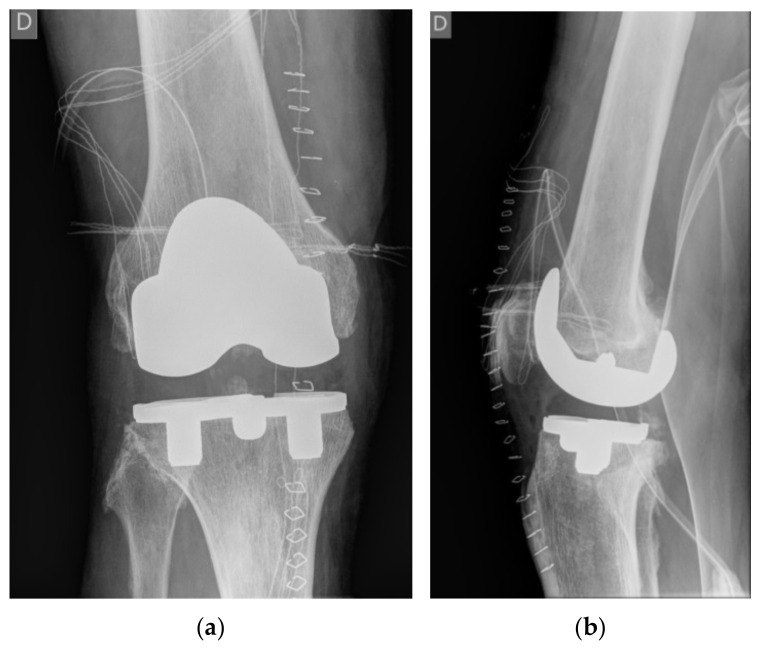
Anteroposterior (**a**) and lateral (**b**) radiographs of the right knee on the day of surgery after inserting the total uncemented endoprosthesis with modular porous tantalum tibial tray.

The patient was a 64-year-old physically active Caucasian man (weight = 88 kg, body mass index (BMI) = 27.8 kg/m^2^) with advanced varus right knee OA who had a primary knee replacement through a medial parapatellar approach with a commercially available uncemented system (NexGen©, Trabecular Metal™ LPS-Flex, Zimmer, Inc., Warsaw, IN, USA). The uncemented components were chosen because the bone quality was high, and a particularly dense sclerotic bone was found at the medial tibial plateau (Figure 1). A size E femoral component, a size 5 Trabecular Metal™ modular tibial component, and a 14 mm high shape-moulded UHMWPE posterior stabilised insert was implanted by an experienced arthroplasty surgeon in a tertiary centre (IN). The patient was instructed to walk with crutches for 6 weeks, and full weight bearing was allowed afterwards.

**Figure 2:**
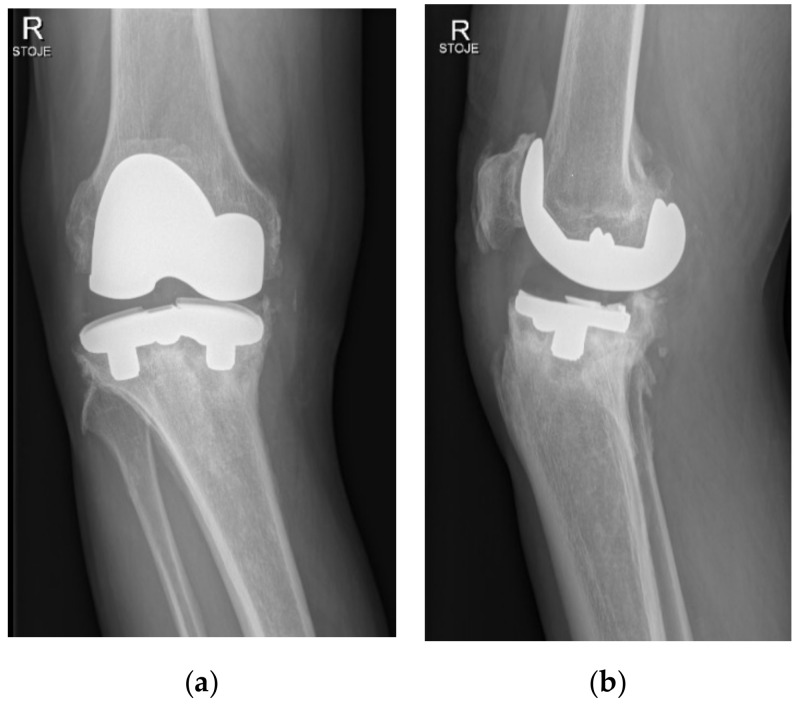
Anteroposterior (**a**) and lateral (**b**) standing radiographs 3.5 years after index operation depicting fracture and displacement of the medial part of the tibial tray.

Two years after surgery, the patient began to experience increasing medial-sided right knee pain during walking without any injury. Then, 3.5 years after primary surgery, the patient was presented at our office because of sudden worsening of pain, varus deformity, and inability to bear weight. Radiographs of the right knee have shown a substantial medial-sided tibial bone loss and tibial tray fracture (Figure 2). Infection was ruled out via standard laboratory tests and right knee aspiration including cell count, Gram stain, and cultures, all of which were negative. The patient was scheduled for a prompt surgical revision.

**Figure 3:**
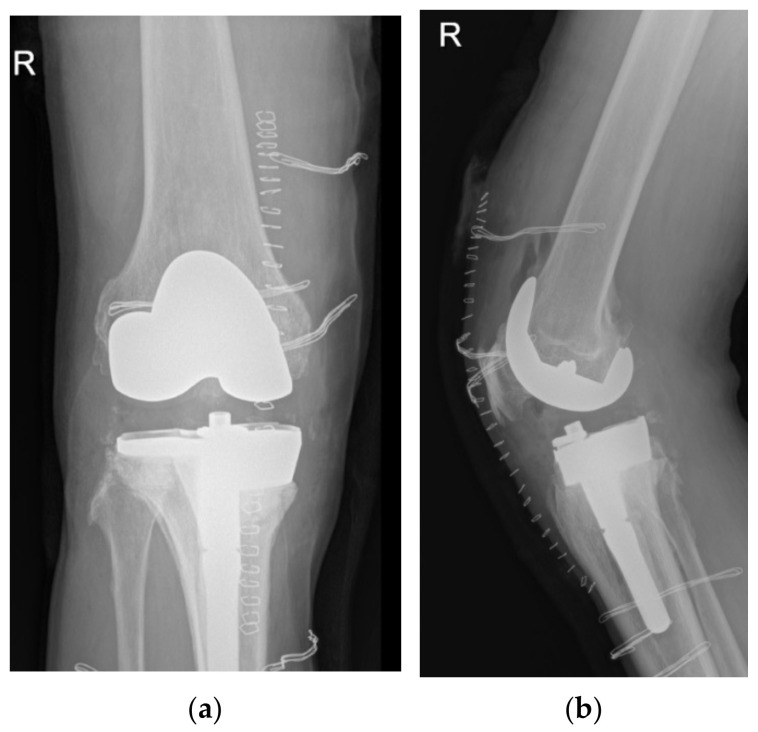
Anteroposterior (**a**) and lateral (**b**) postoperative right knee radiographs depicting the revision total knee construct.

At revision, the tibial baseplate was found completely fractured with the medial part loose over a large proximal medial tibial bone defect, while the femoral component was still stable and undamaged. Soft tissue samples from three different locations were sent for aerobic and anaerobic cultures, and the removed UHMWPE insert was sent for sonication. All samples remained sterile after 14 days of incubation. The broken tibial tray was removed, and the tissue underneath was sent for histologic analysis. Medial-sided structural support for the stemmed cemented revision tibial component was provided through a 15 mm medial tibial augment block. Since no signs of medial collateral ligament laxity were found, the varus/valgus unconstrained 17 mm high (PS UHMWPE) liner was inserted and the wound was closed in layers (Figure 3). The patient followed a standard postoperative course and experienced no perioperative or early postoperative complications.

**Figure 4:**
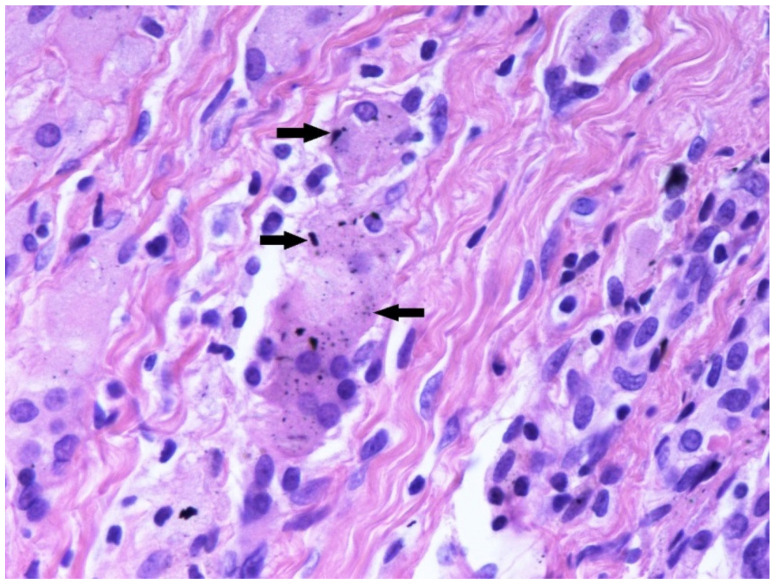
Tissue sample taken underneath the medial tibial baseplate at the revision surgery. Note dark-coloured metallic debris (black arrows). Haematoxylin and eosin (HE) × 400.

Histological examination of tissue underneath the broken part of the tibial baseplate revealed dark-stained metal debris intracellular and in the surrounding area of the multinuclear giant cells suggesting lively phagocytosis (Figure 4).

**Figure 5:**
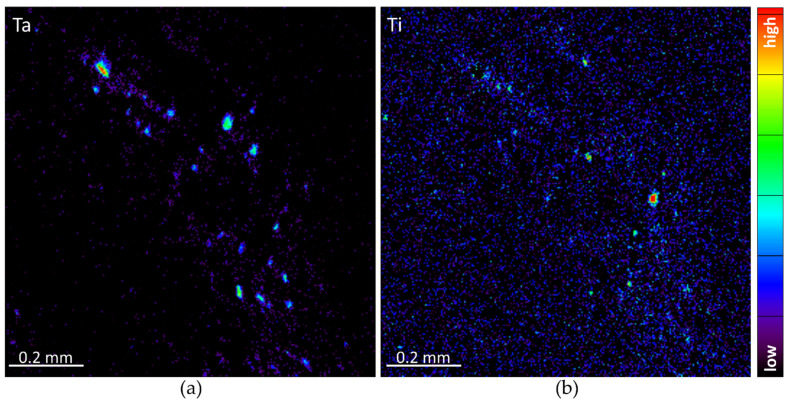
Elemental micro-PIXE maps showing the distribution, in the same scanned area 1 × 1 mm^2^, of Ta (**a**), and Ti (**b**) debris from the degraded tibial tray released into the periprosthetic tissue slice. Elemental maps have been obtained with GeoPIXE 7.5+ software.

Micro-PIXE elemental analysis confirmed the presence of metallic debris from the degraded tibial tray inside the analysed tissue slices. Figure 5 shows the elemental distribution of Ta and Ti obtained with software GeoPIXE 7.5+ from a scanned tissue area of 1 mm^2^. The elemental maps show the large extended area containing metallic debris and the size distribution of debris up to 50 µm per side for some Ta and Ti agglomerates.

**Figure 6:**
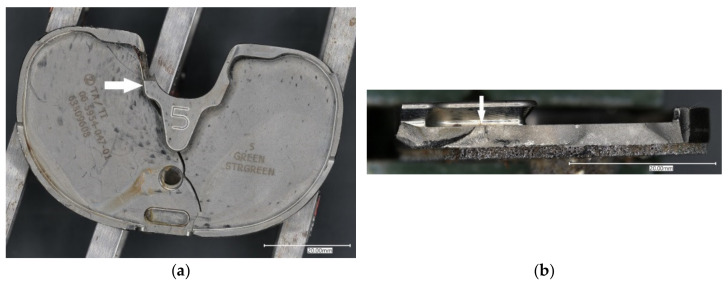
Microphotographs depicting signs of a fatigue fracture of the tibial tray with fatigue crack initiation site (arrows) viewed from above (**a**) and from the fracture side (**b**).

Fractographic analysis showed that the failure of the tibial baseplate fulfilled the criteria of a typical fatigue fracture. The fracture was initiated at the postero-lateral part of the posterior cut-out for the posterior cruciate ligament (PCL) and followed an antero-medial direction through the weakest level part of the baseplate between the medial hexagonal peg and the central housing for the lock-down screw (Figure 6).

**Figure 7:**
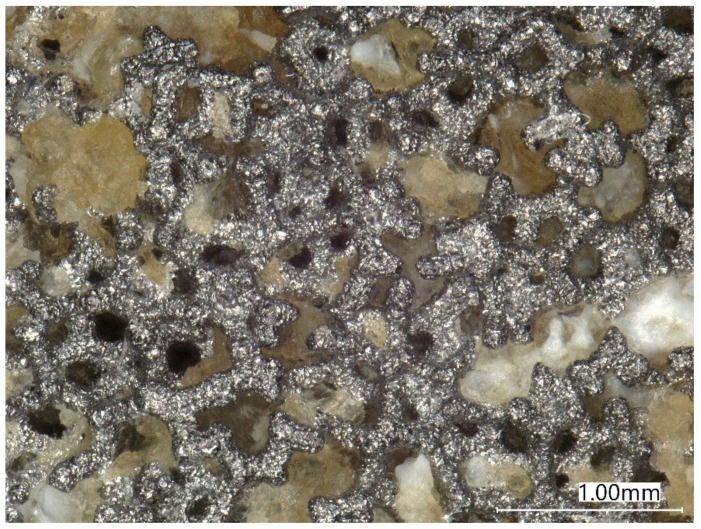
Light microscopy image of the unstained porous tantalum modular tibial tray surface with mostly fibrous tissue (yellow areas) and some bone (white areas) ingrowth.

**Figure 8:**
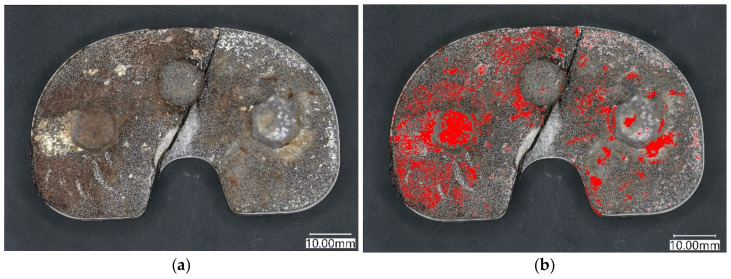
Light microscopy image of fractured modular tray with predominantly fibrous tissue ingrowth (**a**) converted to colour (**b**) showing bone area/pore area (bone area is shown in red). Note less dense bone ingrowth to the medial part of the modular tibial tray.

Macroscopic examination and microstructural analysis of the tibial baseplate contact surface revealed signs of bone ingrowth to the holes of porous tantalum (Figure 7). However, the bone filled only 22.5% of the surface and was even less pronounced in the medial half of the tibial baseplate, suggesting that the bone–implant contact was neither stable enough to provide bone ingrowth nor durable enough to withstand the loads of daily living (Figure 8).

**Figure 9:**
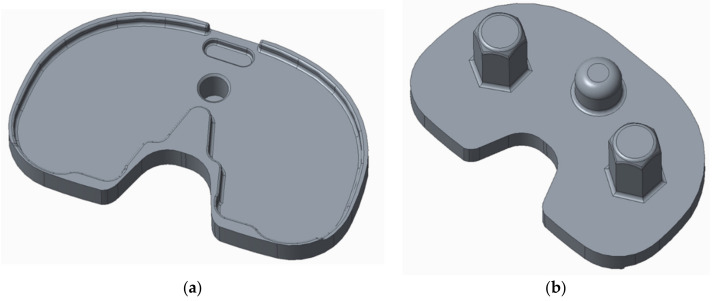
Numerical model of the tibial tray viewed from above (**a**) and from below (**b**).

**Figure 10:**
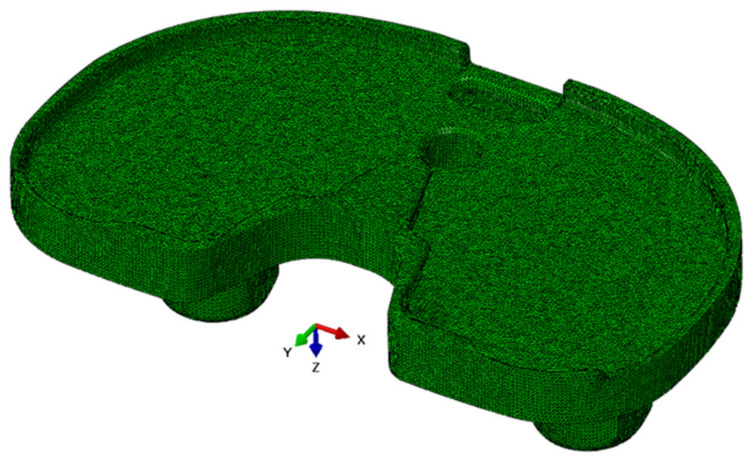
Structural mesh of finite elements of tibial baseplate.

**Figure 11:**
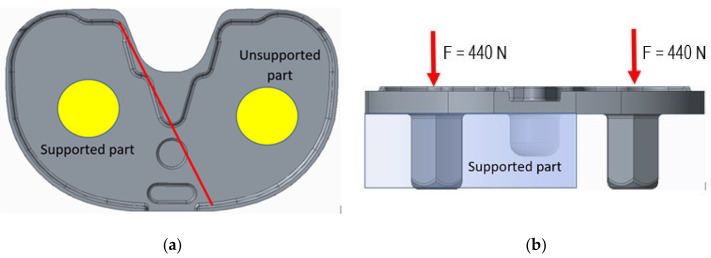
Loading conditions of tibial baseplate in (**a**) top view and (**b**) side view.

**Figure 12:**
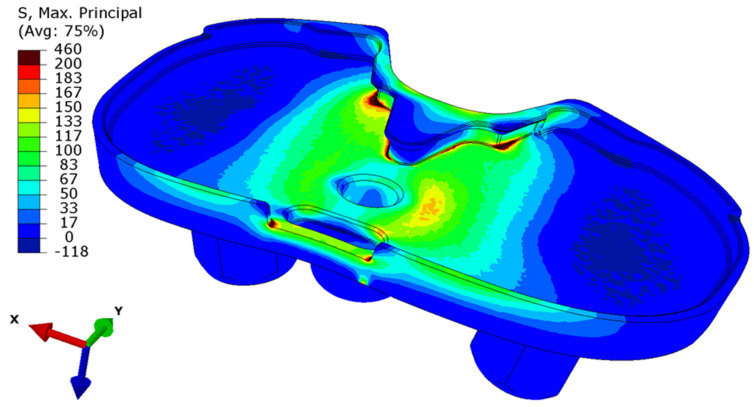
Distribution of maximal principal stresses (in MPa) on the surface of the medially unsupported tibial plate loaded by the patient’s body weight during standing on one leg.

FE modelling allowed the assessment of load distribution on the tibial tray. Figure 9 shows the numerical model of the tibial tray and Figure 10 shows the mesh of FE. The loading conditions of two vertical forces on the tibial baseplate are shown in Figure 11. The red line separates the supported and unsupported part of the tibial baseplate. Yellow circular areas are aligned with the position of the hexagonal plugs and indicate the surface where half of the patient’s weight is uniformly distributed on the tibial baseplate, i.e., when the patient was standing on his right leg. The medial half of the tibial tray was left unsupported. This way, we were able to simulate the actual bone loss of the medial tibial condyle underneath the medial part of the tibial tray. High stress concentration was found at the postero-lateral part of the posterior cut-out for the PCL under the sledge to engage the UHMWPE insert—just where the fatigue fracture was initiated (Figure 12). Maximal principal stress on the surface of the FE solid model without bone support of the medial part of the tibial plate reached 460 MPa. Hence, the dynamic strength of the material could be exceeded and a crack, which later progressed to evident fracture, initiated. Maximum principal stresses are relevant for fatigue crack propagation if the actual stress of the material does not overcome its yield stress.

Obtained tensile mechanical properties were typical for a medical-grade Ti-alloy and were as follows: the tensile yield stress (Rp) was 930 to 970 MPa, the ultimate tensile strength (UTM) was 970 to 997 MPa with a maximum elongation up to 2.3%, Young’s modulus (E) was 124 GPa, and Poisson’s ratio (ν) was 0.3.

## 4. Discussion

The potential advantages of highly porous metallic surfaces consist of high initial fixation and bone stock retention by diminished stress shielding. Porous tantalum has been increasingly used for different orthopaedic applications due to favourable elastic modulus, high coefficient of friction, exceptional porosity, and proven bone ingrowth [17]. It has been shown that porous tantalum preserves bone under the monoblock tibia trays better than cemented implants and in the parallel fashion to the nonoperative control limb [18,19]. In a meta-analysis of porous tantalum monoblock versus cemented tibia in primary TKA, the authors described a slightly higher functional score, fewer radiolucent lines, and shorter operation time in favour of uncemented implant [20]. However, at 5-year follow-up, the authors noted no significant difference regarding the range of motion, Western Ontario and McMaster University Osteoarthritis Index (WOMAC), total complications, reoperation, and loosening of the tibial component between the two groups and [20]. In a long-term study on periprosthetic bone mineral density (BMD), the porous tantalum tibial component was not found to have any favourable effect on the proximal tibia bone loss after TKA [21].

In concordance with Scully et al., we have also found incomplete porous tantalum bone ingrowth and marked bone loss underneath the medial part of the modular tibial tray that resulted in catastrophic failure with the fracture line taking the same path as previously described [13]. The authors linked the causes of this modular posterior stabilised (PS) implant failure to asymmetric and excessive forces across the implant because of component mispositioning, suboptimal sizing and elevated patient body weight [13]. An earlier clinical study has also shown cases of subsidence of PS porous tantalum monoblock tibia trays linked to specific patient factors, i.e., overweight and/or overload [22]. Hanzlik et al. found that the retrieved modular tibial trays had a higher extent of bone ingrowth (29 ± 13%) than the monoblock trays (22 ± 18%), but their results were based on the analysis of only three modular trays revised for stiffness, pain, and infection [12]. In short, the cause of this particular implant failure is currently not known.

The subchondral bone of the tibial plateau at the resection level is the mechanical support for the tibial baseplate of TKA and undergoes bone remodelling in response to changes in mechanical load [23,24]. During level walking on a normal knee, the medial compartment takes over 60–80% of the body weight [25]. It has been shown that the β2-adrenergic receptor (Adrb2) plays a critical role in mechanical stress-induced bone remodelling [26]. Mechanical stress is the main cause of upregulation of Adrb2 in subchondral bone, inducing osteoclast generation and participating in subchondral bone remodelling [27]. Sympathetic nerve fibres were detected in the subchondral bone of OA [28]. Through the excretion of norepinephrine, these fibres are capable of activating the Adrb2, which, in turn, inhibits osteoblast proliferation and differentiation and promotes osteoclast precursor maturation and bone resorption activity [29]. In a model of OA of the temporomandibular joint, increased osteoclast activity via Adrb2 caused bone loss following increased mechanical stress [26]. Orthodontic tooth movement proved that mechanical force caused Adrb2 activation in alveolar bone remodelling [30]. The presented patient has had a varus knee alignment ultimately leading to progressive varus knee OA necessitating primary TKA. Even though it is not possible to exactly measure the alignment on short knee images, it is probable that some varus malalignment persisted. Together with the usual medial tightness of the varus OA knees after TKA, these factors could contribute to the increased mechanical stress inducing tibial bone resorption beneath the medial part of the tibial tray. Minoda et al. have recently shown that BMD was significantly decreased in the medial region after primary TKA with a cementless porous tantalum tibial component, and this bone loss was attributed to the medial peg position regarding to the tibial cortex [31].

A comparative study on the medial sclerotic area and the lateral non-sclerotic area of the tibial plateau in patients with knee OA found that the number and activity of osteoclasts in the sclerotic area was significantly higher than that in the non-sclerotic area [32]. The increased activity of osteoclasts in the sclerotic bone indicated that the activity of bone formation and resorption increased in human knee OA, but no coupling was observed. Podlipec et al. have shown micron-sized wear debris in the vicinity of Ti-alloy total hip arthroplasty (THA) responsible for the tissue oxidative stress, resulting in chronic inflammation [33]. Others have raised concerns for potential failure in shoulder arthroplasty because of the development of metallic debris, increasing in both overall incidence and degree of severity over time after porous tantalum glenoid component insertion [34]. Tantalum and titanium debris found in the present study in the tissue underneath the removed tibial baseplate could have induced increased osteoclast activity that further promoted bone absorption, creating a vicious circle ultimately leading to tibial tray fatigue fracture.

The main strength of this study is the attempt to highlight the mechanisms of catastrophic failure of primary TKA with the modular trabecular metal tibial tray that are currently lacking or limited to re-revision procedures, in which porous tantalum augments were used in conjunction with PMMA or autologous bone grafts [35]. Despite the promising insights, this current study has some limitations. To begin with, the patient serum was not analysed for metals consisting of the implanted TKA components. The main reason for that is the inability of the national laboratory for clinical chemistry and biochemistry to determine serum tantalum values. In addition, it has been shown that serum trace element analysis does not represent a reliable parameter in the management of patients with dubious hip implants [36]. Further on, FEA was performed only in static position (standing on one leg) and not also at common activities of daily living when much larger forces appear. Szarek et al. have clearly shown that during level walking and going downstairs, the knee joint force reaches 283% and 410% of body weight, respectively [37]. Finally, bone ingrowth measurements were not studied in concordance with previously described methods [12].

## 5. Conclusions

This study presents a second clinical case report of extensive medial tibia bone loss resulting in modular porous tantalum tibia baseplate fracture after primary total knee arthroplasty. Reasons for this tilting migration were for the first time highlighted by histologic tissue examination, micro-PIXE tissue analysis, and finite-element modelling of the tibial component to provide a better insight for the catastrophic implant failure necessitating early revision.

The extensive medial tibia bone loss may be the reason for the premature fracture. Histological examination of the periprosthetic tissue underneath the removed tibial baseplate showed the presence of particles in the tissue which were identified as dark-coloured metallic debris. The tissue was scanned with a 3 MeV focused proton beam for Proton-Induced X-ray Emission. We have confirmed that the debris were Ti and Ta particles coming from the endoprosthesis. The presence of Ti and Ta debris in tissue under the removed tibial baseplate may have triggered the osteolysis and generated an imbalanced support for the tibial tray. In order to probe this hypothesis, finite element for mechanical analysis was performed, and the results are in agreement with the fractographic and microstructural analysis showing that the failure of the tibial baseplate presents a high stress concentration at the postero-lateral part of the posterior cut-out, just where the fatigue fracture took place.

Further studies showing long-term clinical non-inferiority, absence of unpredictable complications, and economic cost-effectiveness are needed to prove modular porous tantalum cementless mode of fixation reliable for the patient and well-suited for the treating orthopaedic surgeon before it could be labelled as the new gold standard in TKAs.

## Data Availability

Not applicable.
